# Metabolic Flux Analysis of Mitochondrial Uncoupling in 3T3-L1 Adipocytes

**DOI:** 10.1371/journal.pone.0007000

**Published:** 2009-09-10

**Authors:** Yaguang Si, Hai Shi, Kyongbum Lee

**Affiliations:** 1 Department of Biology, Tufts University, Medford, Massachusetts, United States of America; 2 Department of Chemical and Biological Engineering, Tufts University, Medford, Massachusetts, United States of America; Virginia Tech, United States of America

## Abstract

**Background:**

Increasing energy expenditure at the cellular level offers an attractive option to limit adiposity and improve whole body energy balance. *In vivo* and *in vitro* observations have correlated mitochondrial uncoupling protein-1 (UCP1) expression with reduced white adipose tissue triglyceride (TG) content. The metabolic basis for this correlation remains unclear.

**Methodology/Principal Findings:**

This study tested the hypothesis that mitochondrial uncoupling requires the cell to compensate for the decreased oxidation phosphorylation efficiency by up-regulating lactate production, thus redirecting carbon flux away from TG synthesis. Metabolic flux analysis was used to characterize the effects of non-lethal, long-term mitochondrial uncoupling (up to 18 days) on the pathways of intermediary metabolism in differentiating 3T3-L1 adipocytes. Uncoupling was induced by forced expression of UCP1 and chemical (FCCP) treatment. Chemical uncoupling significantly decreased TG content by ca. 35%. A reduction in the ATP level suggested diminished oxidative phosphorylation efficiency in the uncoupled adipocytes. Flux analysis estimated significant up-regulation of glycolysis and down-regulation of fatty acid synthesis, with chemical uncoupling exerting quantitatively larger effects.

**Conclusions/Significance:**

The results of this study support our hypothesis regarding uncoupling-induced redirection of carbon flux into glycolysis and lactate production, and suggest mitochondrial proton translocation as a potential target for controlling adipocyte lipid metabolism.

## Introduction

At the whole body level, obesity results from a chronically positive energy balance. Drug therapies have generally been aimed at reducing energy intake. For example, sibutramine's mode of action is to suppress appetite [1], while orlistat's mode of action is to block nutrient absorption [2]. Unfortunately, neither drug has shown significant long-term efficacy. An alternative approach for improving bodily energy balance is to increase expenditure at the cellular level. The white adipocyte is a logical target, because its hypertrophic growth directly contributes to obesity [3].

In adipocytes, ATP production occurs mainly in the mitochondria through oxidative phosphorylation. The efficiency of oxidative phosphorylation depends on tight coupling between substrate metabolism, electron transport and proton pumping across the inner mitochondrial membrane. In response to cold stress, brown (but not white) adipocytes can loosen this coupling by inducing a mitochondrial proton channel protein (uncoupling protein-1 or UCP1). Situated across the inner membrane of the mitochondria, UCP1 facilitates the reentry of the protons into the mitochondrial matrix. This reentry bypasses ATP synthase, dissipating the chemical potential across the mitochondrial membrane as heat [4]. The impact of UCP1 induction on energy expenditure in white adipocytes has been studied both *in vivo* [5] and *in vitro* [5], [6], [7], [8]. In these studies, increased UCP1 expression significantly correlated with a reduction in adipocyte or adipose tissue triglyceride (TG) content.

Recently, we examined the metabolic basis of TG reduction using a 3T3-L1 cell line that stably expresses UCP1 under the regulation of a tetracycline-responsive promoter [9]. Our results agreed with previous findings that expressing UCP1 in white adipocyte reduces TG accumulation. However, our data did not support an up-regulation of substrate oxidation. Rather, metabolic rate measurements pointed to a down-regulation of lipid synthesis. Based on this previous work, we hypothesized that sustained mitochondrial uncoupling requires the cell to compensate for the decreased oxidation phosphorylation efficiency by up-regulating glycolysis, resulting in a redirection of carbon flux away from lipid synthesis.

In this study, we addressed this hypothesis by analyzing the effects of mitochondrial uncoupling on the pathways of intermediary metabolism in white adipocytes. Mitochondrial uncoupling was established in two ways. In addition to forced UCP1 expression, we also applied a chemical uncoupler, carbonyl cyanide *p*-trifluromethoxyphenyl-hydrazone (FCCP). Similar to UCP1, FCCP acts as a protonophore to facilitate proton translocation across the inner membrane of the mitochondria [10]. Previous studies have reported that short-term FCCP treatment (up to a few hours) acutely stimulates cellular respiration [11], [12], [13], reduces mitochondrial membrane potential [14], and lowers both fatty acid synthesis [15] and lipolysis [16]. A very recent gene expression analysis performed on 3T3-L1 adipocytes showed that long-term FCCP treatment (up to 6 days) lowered the transcript levels of several enzymes involved in fatty acid synthesis [17]. Here, we used a metabolic flux analysis model [18] to compare chemical and protein-mediated uncoupling at the level of metabolic pathway activity.

## Methods

### Materials

3T3-L1 preadipocytes were obtained from ATCC (Manassas, VA). Tissue culture reagents including Dulbecco's Modified Eagle's Medium (DMEM), calf serum (CS), fetal bovine serum (FBS), human insulin, and penicillin/streptomycin were purchased from Invitrogen (Carlsbad, CA). Unless otherwise noted, all other chemicals were purchased from Sigma (St. Louis, MO).

### Cell culture and differentiation

3T3-L1 preadipocytes carrying pRevTRE-UCP1 or pRevTRE-null plasmids were prepared as described previously [9]. Unmodified and stably transfected 3T3-L1 preadipocytes were plated onto 24-well plates and expanded in preadipocyte growth medium consisting of DMEM supplemented with CS (10% v/v), penicillin and streptomycin. Medium was replenished every other day. Two days post-confluence, the cells were induced to differentiate using an adipogenic cocktail (1 µg/ml insulin, 0.5 mM isobutylmethylxanthine, and 1 µM dexamethasone) added to a basal medium (DMEM with 10% FBS and penicillin/streptomycin). After 48 hrs, the first induction medium was replaced with a second induction medium consisting of the basal adipocyte medium supplemented with only insulin. After another 48 hrs, the second induction medium was replaced with the basal adipocyte medium. This basal maintenance medium was replenished every other day during the remainder of the culture experiments, which lasted 4 to 10 additional days (8 to 14 days following the addition of the first induction medium).

### FCCP treatment

A subset of unmodified 3T3-L1 cells were expanded, induced, and maintained in media containing the chemical uncoupler FCCP. The drug was stored as a 10 mM stock solution in 95% ethanol at −20°C. Fresh working solutions were prepared at the time of treatment by diluting the stock solution with growth, induction or basal maintenance medium as appropriate. A preliminary dose response experiment showed that a working concentration of 1 µM did not adversely affect cell growth and viability. This concentration was used in all FCCP experiments of the present study.

### Microscopy

At the indicated time points, cellular morphology was recorded using phase-contrast microscopy (Nikon-US, Melville, NY). Intracellular lipid droplets were visualized by Oil Red O staining [19].

### Real-time RT PCR

Total RNA was isolated using the RNeasy Mini Kit from QIAGEN (Valencia, CA). Reverse transcription was performed on a PTC-100 Programmable Thermal Controller (MJ Research, Waltham, MA) using the High-Capacity cDNA Archive Kit (Applied Biosystems, Foster City, CA) with random primers. The peroxisome proliferator-activated receptor-γ (PPAR-γ) and glycerol-3-phosphate dehydrogenase (GPDH) mRNA and 18S rRNA levels were determined using the TaqMan Gene Expression assay (Applied Biosystems, Foster City, CA). All gene expression data were normalized to the 18S rRNA contents in corresponding samples.

### Oxygen uptake

Dissolved oxygen in the culture medium was measured using a needle-type fiber-optic micro-sensor (MicroxTX3, PreSens GmbH, Regensburg, Germany). Measurement details, including experimental setup (Figure S1), are described in Supplementary Materials S1. The concentration data were related to the oxygen uptake rate (OUR) using a diffusion-reaction model. The measured OUR was used for relative comparisons between the treatment groups and not used for MFA in order to independently evaluate the calculated OUR.

### Metabolite assays

Metabolite measurements were performed both on cell lysates and spent medium samples as described previously [18]. Cells were lysed *in situ* with a 0.1% SDS buffer and sonicated. Immediately after collection, the spent medium samples were cleaned of cell debris by a brief centrifugation step. Free glycerol, TG and FFA were measured using enzymatic assay kits (Sigma). Glucose and lactate concentrations were measured using the methods of Trinder [20] and Loomis [21], respectively. Amino acids were quantified by HPLC (Alliance 2690, Waters, Milford, MA) using fluorescence-based detection following pre-column derivatization of primary or secondary amines with 6-aminoquinolyl-N-hydroxysuccinimidyl-carbamate [22]. Cellular ATP was measured using a luminescence assay kit (Promega, Madison, WI) that is based on the ATP-dependent activity of luciferase. All metabolite data were normalized by the corresponding cell sample DNA content, which was determined with a fluorescence-based assay using either the Hoechst or PicoGreen dye (Invitrogen). The PicoGreen dye method was applied to samples used for the ATP measurements, because the Hoechst dye interfered with the luminescence assay.

### Mitochondrial membrane potential

Mitochondrial membrane potentials in the untreated control and FCCP-treated cells were determined using tetramethylrhodamine ethyl ester (TMRE) as a fluorescent probe (Invitrogen). Cells were incubated with 100 nM TMRE for 30 min at 37°C. After incubation, the cells were rinsed once with pre-warmed Hank's balanced salt solution (HBSS). The culture plates were then immediately placed in a temperature controlled fluorescence plate reader (Gemini EX, Molecular Devices, Sunnyvale, CA). TMRE fluorescence was measured at 549/574 nm excitation/emission. The membrane potential measurements were performed at various times during the glucose withdrawal-addition experiment as indicated in the Results section. The fluorescence reading for each well was normalized by the corresponding total DNA content.

### Stoichiometric model and metabolic flux analysis (MFA)

A stoichiometric network model of adipocyte intermediary metabolism was constructed as described previously [18]. Table S1 provides a detailed list of the reactions and their stoichiometry. Intracellular fluxes were estimated from measurements on metabolite uptake and output rates (exchange fluxes) by solving a constrained non-linear optimization problem with stoichiometric and thermodynamic constraints [23]. Additional details of the flux calculation are presented in Supplementary Materials S1.

### Statistics

Comparisons between two experimental groups were performed using one-way ANOVA. Group means were deemed to be statistically significantly different when p<0.05.

## Results

### Differentiation

**Figure 1:**
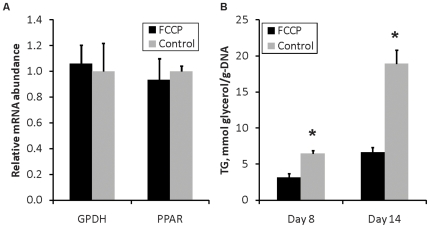
Uncoupling effects on adipogenic conversion. (A) Gene expression levels of GPDH and PPAR-γ on day 8 post-induction. (B) Amount of TG in adipocytes harvested on day 10 post-induction. Data shown are means±SD (*n* = 6). *: significantly different from untreated control (p<0.05).

The chemical uncoupler (FCCP) was first added to the medium 24 hrs following cell seeding and applied throughout the duration of the culture experiments. This protocol was designed to mimic the effects of constitutively expressed uncoupling protein (UCP1). Differentiation was assessed by measuring the mRNA expression of PPARγ and GPDH on day 8 post-induction [24], [25]. Similar to UCP1 [9], FCCP had no statistically significant effect on the expression of either gene (Figure 1A).

### Lipid storage

**Figure 2:**
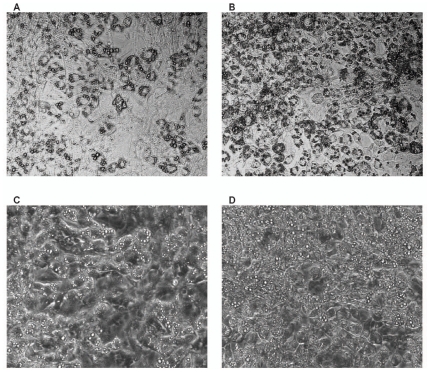
Morphological comparisons. Oil-Red O stained (top row) and phase contrast (bottom row) images of FCCP treated (left column) and untreated control cells (right column) on day 8 post-induction.

The effect of chemical uncoupling on lipid accumulation was assessed through biochemical assays and morphological analysis. Continued treatment with FCCP significantly lowered the amount of accumulated TG by 35% compared to untreated controls (Figure 1B). Oil Red O staining showed that a smaller fraction of the FCCP-treated culture (Figure 2A) contained visible lipid droplets compared to untreated controls (Figure 2B). Phase contrast images (Figures 2C and 2D) suggested that even cells without visible lipid droplets in both treated and untreated control possessed the round shapes of differentiated 3T3-L1 adipocytes.

### Mitochondrial function

**Figure 3:**
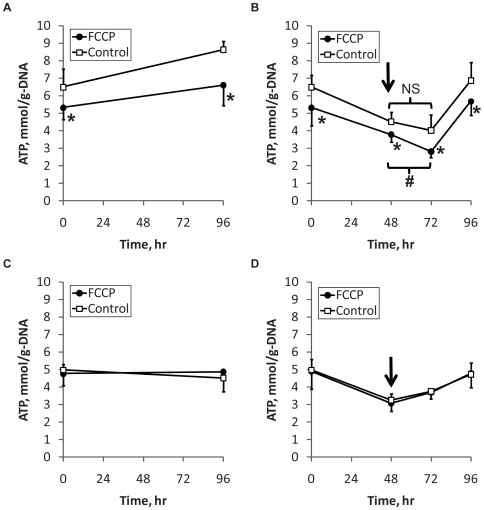
Glucose utilization for ATP production. Effect of long-term (A, B) and short-term chemical uncoupling (C, D) on cellular ATP without (A, C) and with glucose starvation (B, D). In all panels, time zero corresponds to day 10 post-induction. In panels (B) and (D), glucose starvation lasted from time zero to 48 hrs. Cells were again fed glucose-rich (4.5 g/L) maintenance medium at 48 hrs (arrow). In panels (C) and (D), FCCP was first introduced at time zero and replenished with the following medium change at 48 hrs. Data shown are means±SD (n = 6). *: significantly different from the untreated control at the corresponding time point (p<0.05). #: significantly different from the 48 hr time point.

As mitochondrial uncouplers, both UCP1 and FCCP should down-regulate aerobic ATP synthesis and up-regulate oxygen consumption. Previously, we found that forced expression of UCP1 did not significantly affect the ATP level when the cells were incubated in a glucose rich (4.5 g/L) medium (Table S3). However, incubating the UCP1 expressing cells in a glucose free medium significantly depressed the ATP level relative to pRev control cells (Table S3) [9]. Motivated by this finding, the present study assayed the ATP content of FCCP treated and untreated control cells during and after a period of glucose starvation. Unlike UCP1 expression, long-term FCCP treatment significantly decreased the basal ATP level by ca. 18% even without glucose starvation (Figure 3A). Withholding glucose for 48 hrs decreased the ATP levels in the FCCP treated and untreated control cells to similar extents, as the initial difference in the ATP levels between the treatment groups remained nearly unchanged. Following the addition of glucose at the 48 hr time point, the ATP level in the FCCP treated cells continued to decline significantly until the 72 hr time point (Figure 3B). The ATP decline in the untreated control cells leveled off statistically, widening the difference in the ATP levels between the two treatment groups. At the 72 hr time point (24 hrs after reintroducing the glucose rich medium), the ATP level in the FCCP treated cells was 30% lower compared to untreated controls.

In light of the observation that long-term exposure (12 days) to FCCP lowered the ATP level even in the presence of medium glucose, we also examined the effects of short-term exposure. After 2 days, the ATP levels in FCCP treated and untreated control cells were not significantly different when glucose was continuously present (Figure 3C) or withdrawn and then re-introduced (Figure 3D). Similar to long-term FCCP exposure, ATP levels significantly declined in both FCCP treated and control cells during the glucose starvation. Interestingly, the ATP level in the FCCP treated cells halted its decline by the 48 hr time point, rather than the 72 hr time point (Figure 3B). Readings of TMRE fluorescence remained unchanged throughout the duration of the 4 day glucose starvation experiment for both FCCP treated and control cells, indicating that ATP levels could decline without significant alterations in the mitochondrial membrane potential (Figure S2).

**Figure 4:**
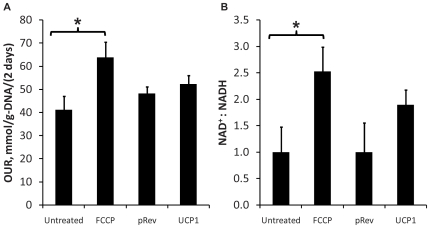
Oxidative metabolism. Effects of forced UCP1 expression or FCCP treatment on (A) oxygen uptake rate (OUR) and (B) cellular NAD^+^:NADH ratio. Data shown are means±SD (*n* = 6). *: significantly different from pRev or untreated control (p<0.05).

A further comparison between the two uncoupling modalities assessed the oxygen uptake rate (OUR), because mitochondrial proton pumping can drive up to 90% of cellular respiration [26]. Measurements on day 10 post-induction showed that the FCCP treatment had significantly increased the OUR by 55% (Figure 4A). Chemical uncoupling also significantly increased the cellular NAD^+^ to NADH ratio by 150% (Figure 4B). The present study repeated the OUR measurements on the UCP1 expressing cells and corresponding controls. The OUR values were not significantly different, consistent with the previous study [9]. Forced expression of UCP1 raised the cofactor ratio, albeit to a smaller and statistically insignificant extent compared to chemical uncoupling.

### Metabolic flux analysis

**Figure 5:**
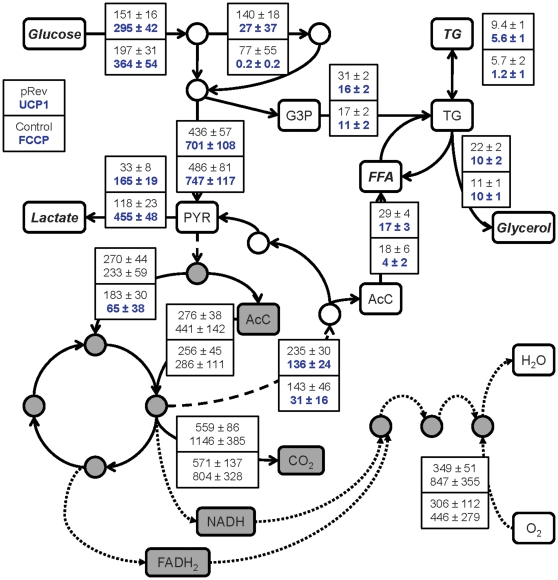
Metabolic flux distribution (day 14 post-induction). Top to bottom, values refer to pRev control, UCP1, untreated control and FCCP, in order. Data shown are means±SD (*n* = 3 for pRev and UCP1; *n* = 6 for untreated control and FCCP). Statistical tests compared UCP1 against pRev and FCCP against untreated control. Significantly different fluxes are shown in bold blue font (p<0.05). Dashed and dotted lines denote electron and inter-compartmental transport steps. Mitochondrial metabolites are shown in grey shaded boxes.

We applied MFA to integrate the metabolite measurements and quantify the broader impact of mitochondrial uncoupling on adipocyte intermediary metabolism. The flux analysis used metabolite data collected between days 12 and 14 post-induction, because the newly differentiated adipocyte were expected to exhibit the mature phenotype by this time [27]. Figure 5 summarizes the estimated fluxes through the major pathways. The complete flux data are shown in Table S2. Overall, there were 18 reactions significantly affected by both UCP1 expression and FCCP treatment. These reactions participated in the following pathways: glycolysis, PPP, lipid metabolism and amino acid metabolism.

UCP1 expression and FCCP treatment increased glucose uptake by 85 and 95%, respectively. Fluxes through reactions in glycolysis increased as well. The increases ranged from 60 to 269% in UCP1 expressing cells and 54 to 129% in FCCP treated cells. The most substantial flux increase involved lactate dehydrogenase (LDH). UCP1 expression and FCCP treatment increased lactate output by 394 and 285%, respectively. Mitochondrial uncoupling had the opposite effect on the PPP. UCP1 expression and FCCP treatment significantly decreased the net flux through PPP by 81 and 100%, respectively.

The flux estimates also pointed to a significant down-regulation of lipogenesis. Phospho*enol*pyruvate carboxykinase (PEPCK) catalyzes the conversion of cytosolic oxaloacetate (OAA) into phospho*enol*pyruvate, a key step in glycerogenesis generating the glycerol moiety of TG. Uncoupling protein-1 expression and FCCP treatment decreased the flux through PEPCK by 36 and 78%, respectively. Uncoupling also reduced the supply of cytosolic acetyl-CoA units for *de novo* fatty acid synthesis. UCP1 expression and FCCP treatment lowered the transport of citrate from the mitochondria into cytosol and its subsequent cleavage into acetyl-CoA and OAA by 42 and 78%, respectively. Overall, UCP1 expression and FCCP treatment depressed the rates of TG formation, accumulation and lipolysis by 40∼52% and 15∼80%, respectively.

The changes in amino acid metabolism were relatively small in magnitude. The largest effects were on the production of proline and alanine. UCP1 expression and FCCP treatment elevated alanine production by 70 and 249%, respectively. Proline formation was nearly zero in the unmodified and untreated control cells. This flux rose to 1.3 mmol/g-DNA/2-days with the long-term FCCP treatment. UCP1 expression increased proline production by 127% compared to the pRev control (transfected with the empty expression plasmid).

## Discussion

A previous study on the metabolic impact of forced UCP1 expression in 3T3-L1 adipocytes indicated a shift of glucose-derived carbon flux into lactate production instead of lipid synthesis [9]. In this study, we mimicked the mitochondrial uncoupling effects of forced UCP1 expression by subjecting cultured 3T3-L1 to mild, but prolonged FCCP treatment. Similar to UCP1 expression, this treatment did not affect the transcript levels of PPARγ or GPDH, two markers of adipocyte differentiation. Chemical uncoupling also reduced the accumulation of TG while increasing glucose uptake and lactate output. Model calculations revealed generally consistent metabolic flux changes for both FCCP- and UCP1-mediated uncoupling, suggesting a common mechanism.

Mitochondrial uncoupling through UCP1 expression and FCCP treatment both down-regulated *de novo* fatty acid synthesis (reaction # 24, Table S2), TG synthesis and storage, and lipolysis (Figure 5). The uncoupling also reduced the fluxes through several reactions in other parts of intermediary metabolism that directly or indirectly couple to fatty acid synthesis. These reactions included steps in the PPP, PEPCK-catalyzed formation of PEP, and citrate lyase (CL)-catalyzed formation of cytosolic acetyl-CoA. In adipocytes, the PPP is a quantitatively important source of NADPH for *de novo* fatty acid synthesis [28]. Not surprisingly, the activity of the PPP depends on the cellular demand for NADPH [29]. This regulatory relationship suggests that the decrease in the PPP flux also reflects a metabolic adjustment to the uncoupling. Similarly, the reduced demand for fatty acid and TG building blocks, respectively, could explain the reduced flux of acetyl-CoA and PEP.

The estimated flux changes were generally consistent with the results of a recent study characterizing the impact of chemical uncoupling on gene expression in mature adipocytes [17]. Expression profiling following short-term (24- or 72-hr) FCCP treatment found decreased transcript levels of a number of lipogenic enzymes, including CL, PEPCK, mitochondrial glycerol-3-phosphate acyltransferase (GPAT), dihydroxyacetone-phosphate acetyltransferase (DHAPAT), fatty acid synthase, HSL and ATGL. In the same study, measurement of *de novo* fatty acid synthesis using tritium labeled acetate estimated a 60% reduction. Model calculations of the present study estimated similar levels of reduction for long-term FCCP treatment and UCP1 expression (77 and 41%, respectively).

Another shared effect of UCP1 expression and FCCP treatment was to substantially elevate net glucose consumption and lactate production. A possible explanation for this flux adjustment is that the prolonged uncoupling elicited a metabolic adaptation to diminished oxidative phosphorylation efficiency. Cultured adipocytes derive ATP primarily from glucose oxidation, because they have only a limited capacity to mobilize intracellular fat stores through β-oxidation [30]. Prior studies by our laboratory and others have shown that neither UCP1 expression nor FCCP treatment significantly stimulate β-oxidation in 3T3-L1 adipocytes [9], [17]. Conversion of glucose into lactate production via glycolysis is an alternative route to ATP generation. While the yield of ATP is less (per mole of glucose), this route does not involve oxidative phosphorylation in the mitochondria. In this regard, mitochondrial uncoupling could induce a greater dependence on glycolytic ATP generation, especially when there is an abundant supply of glucose in the medium. In a previous study [9], we found that ATP levels declined more sharply in the UCP1-expressing adipocytes when medium glucose was removed. In this study, we found that FCCP treatment significantly depressed the ATP level even in the presence of medium glucose, while further increasing the ratio of lactate production to glucose consumption (Figure 5). Other studies on inhibitors of mitochondrial metabolism have similarly noted that glycolytic ATP generation is critical for cell survival when oxidative phosphorylation is impaired [31], [32], [33].

Taken together, the results of this study and other prior works [9], [17], [34] suggest the following link between mitochondrial uncoupling and reduction in TG accumulation. Uncoupling diminishes the efficiency of oxidative phosphorylation, resulting in a lower yield of ATP. In mammalian cells, biosynthesis pathways are among the most sensitive to ATP supply [35]. Sustained over time, the lower ATP yield could signal a down-regulation of lipogenesis at the level of pathway flux, enzyme synthesis, or both.

Down-regulation of lipogenic flux could involve a change in cellular redox state. Long-term treatment with FCCP significantly increased the NAD^+^ to NADH ratio. Forced UCP1 expression elicited a similar trend, although the ratio change was not statistically significant. The NAD^+^ to NADH ratio has been linked to the activities of several metabolic signaling pathway components, including Sirt1, Clock, and CtBP (reviewed in [36]). Another possible regulatory response is that the lower ATP yield stimulated the AMP-activated protein kinase (AMPK) pathway. In adipocytes, AMPK serves as a metabolic master switch [37]. Once stimulated, the AMPK pathway can reduce ATP utilization by inactivating enzymes involved in biosynthesis, including lipogenesis [38]. Elevated AMPK activity has been found in white adipose tissue of ap2-UCP1 transfected mice [39], adenovirus-induced hyperleptinemic rats [6], adiponectin treated rats [40], and rats after exercise [41]. Interestingly, these animal studies also found reduced TG levels in the adipose tissue. The AMPK pathway has been shown to stimulate glucose transport and glycolysis [42], which could explain the up-regulation of glucose uptake and lactate output observed in the present study. On the other hand, AMPK activation by mitochondrial uncoupling was not confirmed in the present study. Establishing a direct role for this and other signaling pathways warrants further studies involving knock down or inhibition experiments.

While the qualitative effects of UCP1 and FCCP were substantially similar, there were quantitative differences. In general, the chemical uncoupler generally elicited larger responses. For example, FCCP treatment increased the OUR by 55%, but UCP1 expression did not result in a significant change. Long-term treatment with FCCP significantly depressed the cellular ATP level even in the presence of high glucose (4.5 g/L) in the culture medium. Forced expression of UCP1 did not significantly depress the ATP level relative to pRev control unless glucose was removed from the medium. These differences may reflect an important distinction between chemical and protein mediated uncoupling. Uncoupling protein activity is subject to regulation through both generic and specific endogenous mechanisms. For example, UCP1 levels may be lowered by proteolytic degradation. Alternatively, its activity may be modulated by free fatty acids. It has been shown that free fatty acids bind and activate UCP1 in brown adipocytes [43]. Finally, FCCP is a synthetic compound whose metabolic fate in the adipocyte remains to be clarified. As the adipocyte does not express high levels of xenobiotic transformation enzymes, it is unlikely that there are endogenous mechanisms to regulate FCCP activity.

The significant reduction in the basal ATP level (before starvation) by long-term FCCP treatment might also explain the relatively minor effect of glucose starvation. Statistical analysis showed that the ATP level declined further between the 48 and 72 hr time points in the FCCP treated cells compared to the control cells. While statistically significant, this decline was modest. One explanation is that the ATP level in the FCCP treated cells was already nearing a lower limit necessary for maintaining cellular viability. Interestingly, treating the adipocytes with the same dose of FCCP (1 µM) for a shorter period of time (up to 4 days) did not adversely affect the ATP level (Figure 3D), suggesting that different metabolic adjustments may occur depending on the duration of the uncoupling stress.

In summary, our results suggest that long-term mitochondrial uncoupling reduces TG accumulation by diverting glucose-derived carbon flux away from fatty acid synthesis into lactate production. The molecular mechanisms underlying these observations remain to be elucidated. In particular, further studies are warranted to determine the mechanisms of sensing and transducing alterations in oxidative phosphorylation efficiency. Additional studies are also needed to explore other mitochondrial membrane components, for example proton carriers, as prospective targets for controlling lipid accumulation in adipocytes.

## Supporting Information

Supplementary Materials S1 Supplementary Methods, Figure Legends and References
(0.14 MB PDF)

Figure S1 Experimental setup for oxygen uptake measurements (not drawn to scale). a: micro-sensor needle; b: dissolved oxygen; c: adipocyte monolayer; d: hot plate. Medium height and needle distance are indicated in the figure.
(0.96 MB TIF)

Figure S2 Mitochondrial membrane potential (MMP). Effect of FCCP treatment without (A) and with (B) glucose starvation. As in Figure 3, time zero corresponds to day 10 post-induction and glucose starvation lasted for 48 hrs (arrow). Cells were again fed glucose-rich (4.5 g/L) maintenance medium at 48 hrs.
(0.90 MB TIF)

Table S1 Reaction stoichiometry of the model adipocyte network
(0.08 MB DOC)

Table S2 Metabolic flux profiles on day 14 post-induction
(0.13 MB DOC)

Table S3 Effect of forced UCP1 expression on cellular ATP
(0.03 MB DOC)
