# Supplementary material for: Metabolic Flux Analysis of Mitochondrial Uncoupling in 3T3-L1 Adipocytes
Source: PLoS One. 2009 Sep 10;4(9):e7000. doi: 10.1371/journal.pone.0007000 (PMC2734990; doi:10.1371/journal.pone.0007000)
Supplement: Supplementary Materials S1 — Supplementary Methods, Figure Legends and References (0.14 MB PDF) [file pone.0007000.s001.pdf]

SUPPLEMENTARY METHODS

Oxygen uptake - Dissolved oxygen in the culture medium was measured using a needle-type fiber-optic micro-sensor (MicroxTX3, PreSens GmbH, Regensburg, Germany). On the day of a measurement, the culture medium was replaced by basal adipocyte maintenance medium buffered with 10 mM HEPES. The micro-sensor was carefully positioned over the culture plate using a micro-manipulator such that the tip was immersed in the medium without touching the bottom of the plate (Figure S1). A hot plate was used to maintain the medium temperature at 37°C.

The concentration data were related to the oxygen uptake rate (OUR) using a diffusion-reaction model, assuming zero convection and constant diffusivity and fluid properties:

$$\frac{d^2c}{dz^2} = 0 \qquad \text{(S1)}$$

$$\text{B.C. 1: } c(z = 0) = c_0 \qquad \text{(S2)}$$

$$\text{B.C. 2: } W(z = -h_b) = -D_{O_2}\frac{dc}{dz}\bigg|_{z=-h_b} = J_{O_2} \qquad \text{(S3)}$$

where c_0 and h_b are the oxygen concentration at medium surface and height of the medium above the bottom of the culture well, respectively. The position coordinate z is defined with respect to the medium surface as the origin such that the bottom of the culture plate (and cell monolayer) is at $z=-h_b$. The second boundary condition (equation S3) equates the molar flux of oxygen at the bottom of the culture well to the rate of oxygen consumption by the cell per unit area. Integrating equation S1, applying the boundary conditions, and rearranging, we obtain:

$$J_{O_2} = \frac{D_{O_2}}{h_m}(c_0 - c_m) \qquad \text{(S4)}$$

where h_m and c_m are the distance between the probe and the medium surface and the measured oxygen concentration in the medium (with cells) at the probe depth. The reference concentration c_0 is determined by measuring the oxygen concentration under the cell-free condition. Finally, the OUR is calculated by dividing the molar flux J_{O2} by the cell density as measured by the total DNA content of the culture well.

Stoichiometric model – A stoichiometric model of adipocyte intermediary metabolism was constructed as follows. First, mouse specific lists of enzyme-mediated reactions were collected from an annotated genome database [1]. Second, stoichiometric information was added for each of the collected enzymes by cross-referencing their common names and enzyme commission names [2]. Third, biochemistry textbooks [3,4] and the published literature [5] were consulted to eliminate enzymes thought to be inactive in the fed state. Finally, the following assumptions were applied: glycogen synthesis is negligible [6]; fatty acid oxidation is small compared to both lipogenesis and lipolysis [7]; the pentose phosphate pathway (PPP) is operating in the oxidative mode [8]; and net protein synthesis is small compared to metabolic fluxes [9]. The stoichiometric model was rendered into a compound, directed graph, visualized using the Bioinformatics toolbox of MATLAB (MathWorks, Natick, MA), and corrected for missing steps and nonsensical dead ends. Reversible reactions flanked by irreversible reactions were assigned directionality so as to ensure unidirectional metabolic flux between the flanking reactions. The final adipocyte model consisted of the following pathways: anaplerosis, glycolysis,

glycerogenesis, ketone body synthesis, lipogenesis, lipolysis, the malate cycle, the PPP, and the tricarboxylic acid (TCA) cycle (Table S1).

Flux calculation - Intracellular fluxes were estimated by solving a constrained non-linear optimization problem as described previously [10]:

$$\text{Min:} \quad \sum_k \left(v_k - v_k^{obs}\right),\ k \in \{\text{external fluxes}\} \tag{S5}$$

$$\text{Subject to:} \quad \boldsymbol{S} \cdot \boldsymbol{v} = \boldsymbol{0} \tag{S6}$$

$$\boldsymbol{G} \cdot \boldsymbol{v} \leq \boldsymbol{0} \tag{S7}$$

where the objective is to minimize the sum squared error between experimentally observed and calculated exchange fluxes. Equation S6 expresses the balances around intracellular metabolites using an $M \times N$ stoichiometric matrix S and an $N \times 1$ steady-state flux distribution vector $\boldsymbol{v}$. Inequality S7 expresses constraints derived from the Gibbs free energy change (ΔG) form of the Second Law.

SUPPLEMENTARY FIGURE LEGENDS

Figure S1. Experimental setup for oxygen uptake measurements (not drawn to scale). a: micro-sensor needle; b: dissolved oxygen; c: adipocyte monolayer; d: hot plate. Medium height and needle distance are indicated in the figure.

Figure S2. Mitochondrial membrane potential (MMP). Effect of FCCP treatment without (A) and with (B) glucose starvation. As in Figure 3, time zero corresponds to day 10 post-induction and glucose starvation lasted for 48 hrs (arrow). Cells were again fed glucose-rich (4.5 g/L) maintenance medium at 48 hrs.

SUPPLEMENTARY REFERENCES

1. Ma H, Zeng AP (2003) Reconstruction of metabolic networks from genome data and analysis of their global structure for various organisms. Bioinformatics 19: 270-277.

2. Kanehisa M, Goto S, Hattori M, Aoki-Kinoshita KF, Itoh M, et al. (2006) From genomics to chemical genomics: new developments in KEGG. Nucleic Acids Res 34: D354-357.

3. Lehninger AL, Nelson DL, Cox MM (2005) Principles of biochemistry. New York: W.H. Freeman. (various pagings) p.

4. Stryer L (1995) Biochemistry: W.H. Freeman

5. Fell DA, Small JR (1986) Fat synthesis in adipose tissue. An examination of stoichiometric constraints. Biochem J 238: 781-786.

6. Kather H, Rivera M, Brand K (1972) Interrelationship and control of glucose metabolism and lipogenesis in isolated fat-cells. Effect of the amount of glucose uptake on the rates of the pentose phosphate cycle and of fatty acid synthesis. Biochem J 128: 1089-1096.

7. Wang T, Zang Y, Ling W, Corkey BE, Guo W (2003) Metabolic Partitioning of Endogenous Fatty Acid in Adipocytes. Obesity Research 11: 880-887.

8. Cabezas H, Raposo RR, Melendez-Hevia E (1999) Activity and metabolic roles of the pentose phosphate cycle in several rat tissues. Mol Cell Biochem 201: 57-63.

9. Herrera MG (1965) Amino acid and protein metabolism. In: Renold A.E.; George F. Cahill J, editor. Handbook of physiologyAdipose Tissue. Washington, DC: American Physiological Society. pp. 382.

10. Nolan RP, Fenley AP, Lee K (2006) Identification of distributed metabolic objectives in the hypermetabolic liver by flux and energy balance analysis. Metab Eng 8: 30-45.
