# Supplementary figures and images for: Metabolic Flux Analysis of Mitochondrial Uncoupling in 3T3-L1 Adipocytes
Source: PLoS One. 2009 Sep 10;4(9):e7000. doi: 10.1371/journal.pone.0007000 (PMC2734990; doi:10.1371/journal.pone.0007000)

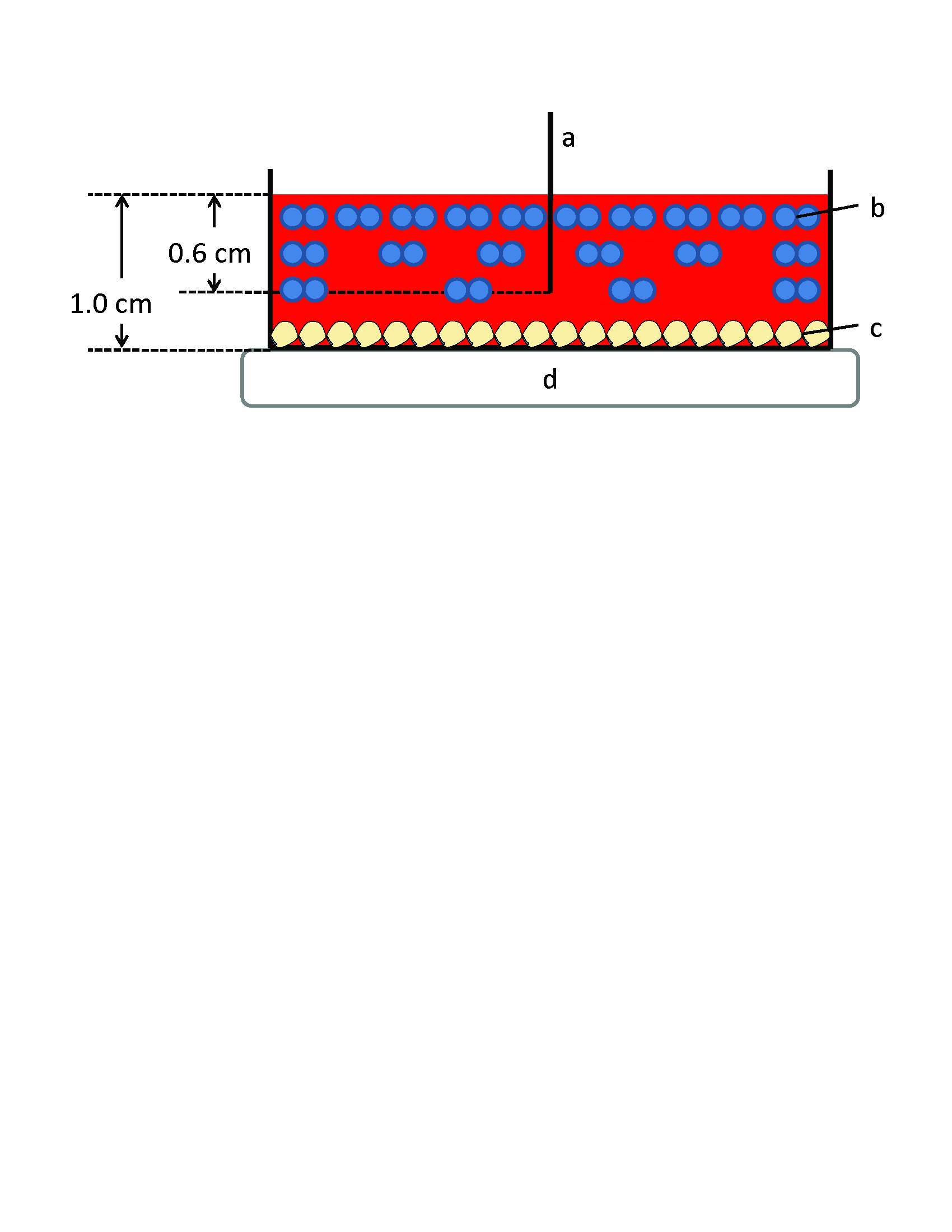

Supplement: Figure S1 — Experimental setup for oxygen uptake measurements (not drawn to scale). a: micro-sensor needle; b: dissolved oxygen; c: adipocyte monolayer; d: hot plate. Medium height and needle distance are indicated in the figure. (0.96 MB TIF) [file pone.0007000.s002.tif]

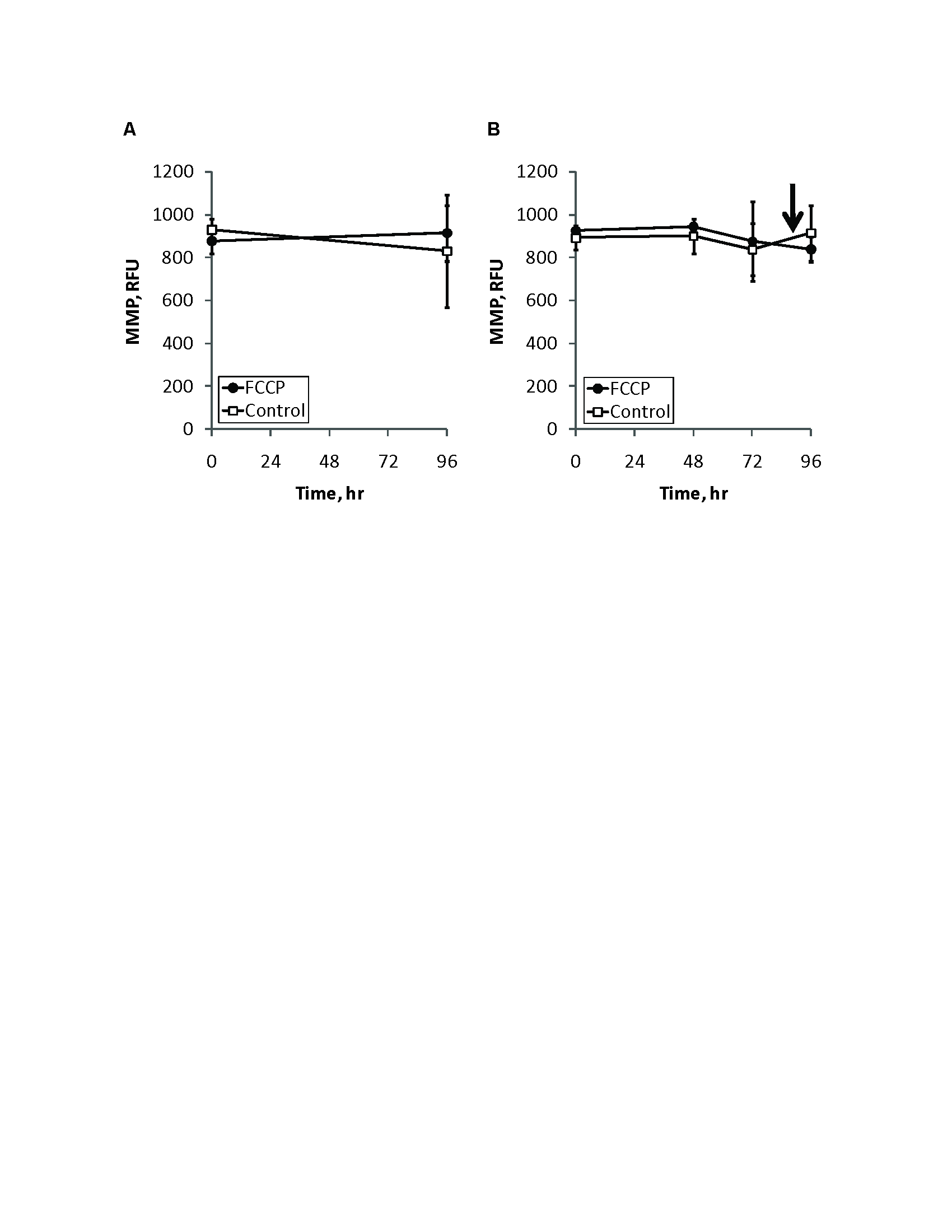

Supplement: Figure S2 — Mitochondrial membrane potential (MMP). Effect of FCCP treatment without (A) and with (B) glucose starvation. As in Figure 3, time zero corresponds to day 10 post-induction and glucose starvation lasted for 48 hrs (arrow). Cells were again fed glucose-rich (4.5 g/L) maintenance medium at 48 hrs. (0.90 MB TIF) [file pone.0007000.s003.tif]
