# Supplementary material for: Metabolic Flux Analysis of Mitochondrial Uncoupling in 3T3-L1 Adipocytes
Source: PLoS One. 2009 Sep 10;4(9):e7000. doi: 10.1371/journal.pone.0007000 (PMC2734990; doi:10.1371/journal.pone.0007000)
Supplement: Table S1 — Reaction stoichiometry of the model adipocyte network (0.08 MB DOC) [file pone.0007000.s004.doc]

**Table S1**. Reaction stoichiometry of the model adipocyte network

| **No.** | **Pathway** | **Stoichiometry** |
| --- | --- | --- |
| 1 | Glycolysis | **Glucose** + ATP = Glucose 6-P + ADP |
| 2 | Glycolysis | Glucose 6-P = Fructose 6-P |
| 3 | Glycolysis | Fructose 6-P + ATP = Glyceraldehyde 3-P + Glycerone-P + ADP |
| 4 | Glycolysis | Glycerone-P = Glyceraldehyde 3-P |
| 5 | Glycolysis | Glyceraldehyde 3-P + NAD+ + ADP + Pi = P-Enolpyruvate + ATP + H2O |
| 6 | Glycolysis | P-Enolpyruvate + ADP + H+ = Pyruvate + ATP |
| 7 | Glycolysis | Pyruvate + NADH + H+ = **Lactate** + NAD+ |
| 8 | Pentose phosphate pathway | Glucose 6-P + 2 NADP+ + H2O = Ribulose 5-P + CO2 + 2 NADPH + 2 H+ |
| 9 | Pentose phosphate pathway | 3 Ribulose 5-P = 2 Fructose 6-P + Glyceraldehyde 3-P |
| 10 | TCA cycle (mitochondria) | ***Pyruvate*** + ***Oxaloacetate*** + NAD+ + H2O = ***Citrate*** + CO2 + NADH + H+ |
| 11 | TCA cycle (mitochondria) | ***Pyruvate*** + HCO3- + ATP = ***Oxaloacetate*** + ADP + Pi |
| 12 | TCA cycle (mitochondria) | ***Citrate*** + NAD+ = ***2-Oxoglutarate*** + CO2 + NADH + H+ |
| 13 | TCA cycle (mitochondria) | ***2-Oxoglutarate*** + NAD+ + CoA = ***Succinyl-CoA*** + CO2 + NADH |
| 14 | TCA cycle (mitochondria) | ***Succinyl-CoA*** + FAD + Pi + ADP = ***Fumarate*** + FADH2 + ATP + CoA |
| 15 | TCA cycle (mitochondria) | ***Fumarate*** + H2O = ***Malate*** |
| 16 | TCA cycle (mitochondria) | ***Malate*** + NAD+ = ***Oxaloacetate*** + NADH + H+ |
| 17 | TCA cycle | Citrate + CoA + ATP = Acetyl-CoA + Oxaloacetate + ADP + Pi |
| 18 | TCA cycle | Oxaloacetate + NADH + H+ = Malate + NAD+ |
| 19 | TCA cycle | Malate + NADP+ = Pyruvate + CO2 + NADPH |
| 20 | TCA cycle | Citrate + NADP+ = 2-Oxoglutarate + CO2 + NADPH + H+ |
| 21 | TCA cycle | Oxaloacetate + ATP = P-Enolpyruvate + CO2 + ADP |
| 22 | Oxidative phosphorylation | NADH + 0.5 O2 + 3 ADP + 3 Pi + 4 H+ = NAD+ + 3 ATP + 4 H2O |
| 23 | Oxidative phosphorylation | FADH2 + 0.5 O2 + 2 ADP + 2 Pi + 3 H+ = FAD + 2 ATP + 3 H2O |
| 24 | Palmitate biosynthesis | 8 Acetyl-CoA + 14 NADPH + 7 ATP + 7 HCO3- + 14 H+ = Palmitate + 14 NADP+ + 8 CoA + 7 ADP + 7 Pi + 7 CO2 + 6 H2O |
| 25 | Tripalmitoylglycerol biosynthesis | Glycerone-P + 3 Palmitate + NADH + 3 ATP + H2O + H+ = Tripalmitoylglycerol + NAD+ + Pi + 3 AMP + 3 PPi |
| 26 | Tripalmitoylglycerol biosynthesis | Tripalmitoylglycerol + 3 H2O = **Glycerol** + 3 Palmitate |
| 27 | Tripalmitoylglycerol accumulation | Tripalmitoylglycerol = **Tripalmitoylglycerol** |
| 28 | Metabolism of ketone bodies | 2 ***Acetyl-CoA*** = ***Acetoacetate*** + 2 CoA |
| 29 | Metabolism of ketone bodies | ***Acetoacetyl-CoA*** = ***Acetoacetate*** + CoA |
| 30 | Metabolism of ketone bodies | ***Acetoacetate*** + NADH = **3-Hydroxybutyrate** |
| 31 | Amino acid metabolism | Pyruvate + NH4+ + NADPH = Alanine |
| 32 | Amino acid metabolism | Aspartate + NH4+ = **Asparagine** |
| 33 | Amino acid metabolism | Aspartate = Oxaloacetate + NH4+ + NADH |
| 34 | Amino acid metabolism | Cysteine = Pyruvate + NH4+ + NADH |
| 35 | Amino acid metabolism | Glutamate = 2-Oxoglutarate + NH4+ + NADH |
| 36 | Amino acid metabolism | Glutamate + NH4+ + ATP = **Glutamine** |
| 37 | Amino acid metabolism | Serine + THF = Glycine |
| 38 | Amino acid metabolism | **Histidine** + THF = Glutamate + NH4+ |
| 39 | Amino acid metabolism | **Isoleucine** + 2 CoA = ***Succinyl-CoA*** + ***Acetyl-CoA*** + NH4+ + FADH2 + 2 NADH |
| 40 | Amino acid metabolism | **Leucine** + CoA + CO2 + ATP = ***Acetoacetate*** + ***Acetyl-CoA*** + NH4+ + FADH2 + 2 NADH |
| 41 | Amino acid metabolism | **Lysine** = 2-Oxoadipate + 2 NH4+ + 3 NADH |
| 42 | Amino acid metabolism | 2-Oxoadipate + CoA = ***Acetoacetyl-CoA*** + 2 CO2 + FADH2 + 2 NADH |
| 43 | Amino acid metabolism | **Methionine** + Serine + ATP + CoA + THF = ***Succinyl-CoA*** + Cysteine + NH4+ + NADH |
| 44 | Amino acid metabolism | **Phenylalanine** + O2 + NADH = Tyrosine |
| 45 | Amino acid metabolism | Glutamate + ATP + 2 NADPH = **Proline** |
| 46 | Amino acid metabolism | Serine = Pyruvate + NH4+ |
| 47 | Amino acid metabolism | **Threonine** + CoA = Glycine + ***Acetyl-CoA*** + NADH |
| 48 | Amino acid metabolism | **Tryptophan** + 3 O2 + NADPH = 2-Oxoadipate + Alanine + CO2 + NH4+ |
| 49 | Amino acid metabolism | Tyrosine + 2 O2 = ***Acetoacetate*** + ***Fumarate*** + CO2 + NH4+ + NADH |
| 50 | Amino acid metabolism | **Valine** + CoA = ***Succinyl-CoA*** + CO2 + 4 NADH + FADH2 + NH4+ |
| 51 | Plasma exchange | Palmitate = **Palmitate** |
| 52 | Plasma exchange | ***Acetoacetate*** = **Acetoacetate** |
| 53 | Plasma exchange | Alanine = **Alanine** |
| 54 | Plasma exchange | **Aspartate**  = Aspartate |
| 55 | Plasma exchange | **Cysteine** = Cysteine |
| 56 | Plasma exchange | **Glutamate =** Glutamate |
| 57 | Plasma exchange | Glycine = **Glycine** |
| 58 | Plasma exchange | **Serine** = Serine |
| 59 | Plasma exchange | **Tyrosine** = Tyrosine |
| 60 | Plasma exchange | **O2 =** O2 |
| 61 | Plasma exchange | CO2 = **CO2** |
| 62 | Plasma exchange | NH4+ =  **NH4+** |
| 63 | Mitochondrial exchange | Pyruvate = ***Pyruvate*** |
| 64 | Mitochondrial exchange | ***Citrate*** + Malate = Citrate + ***Malate*** |
| 65 | Mitochondrial exchange | 2-Oxoglutarate +  ***Malate*** = ***2-Oxoglutarate*** + Malate |
| 66 | Mitochondrial exchange | ***Malate*** + Pi = Malate + ***Pi*** |

Extracellular metabolites are indicated in bold. Mitochondrial metabolites are indicated in bold italics. Several entries in the table (e.g. reaction #10) represent pseudo-reactions obtained by condensing sequences of non-branching reactions.
