# Supplementary material for: Metabolic Flux Analysis of Mitochondrial Uncoupling in 3T3-L1 Adipocytes
Source: PLoS One. 2009 Sep 10;4(9):e7000. doi: 10.1371/journal.pone.0007000 (PMC2734990; doi:10.1371/journal.pone.0007000)
Supplement: Table S2 — Metabolic flux profiles on day 14 post-induction (0.13 MB DOC) [file pone.0007000.s005.doc]

**Table S2**. Metabolic flux profiles on day 14 post-induction

| **No.** | **pRev** | | | **UCP1** | | | | **Untreated control** | | | **FCCP** | | | |
| --- | --- | --- | --- | --- | --- | --- | --- | --- | --- | --- | --- | --- | --- | --- |
| 1 | 151.0 | ± | 15.8 | 295.0 | ± | 41.8 | * | 196.8 | ± | 31.2 | 364.1 | ± | 53.8 | * |
| 2 | 10.5 | ± | 4.7 | 267.7 | ± | 77.2 | * | 119.6 | ± | 61.9 | 363.9 | ± | 54.0 | * |
| 3 | 104.2 | ± | 10.0 | 285.9 | ± | 53.4 | * | 171.1 | ± | 35.5 | 364.0 | ± | 53.8 | * |
| 4 | 73.1 | ± | 10.7 | 269.9 | ± | 54.1 | * | 154.1 | ± | 36.9 | 353.3 | ± | 53.1 | * |
| 5 | 224.2 | ± | 26.2 | 564.9 | ± | 95.7 | * | 350.9 | ± | 65.3 | 717.3 | ± | 106.8 | * |
| 6 | 436.6 | ± | 57.3 | 700.6 | ± | 107.5 | * | 485.9 | ± | 80.9 | 747.0 | ± | 117.4 | * |
| 7 | 33.4 | ± | 7.6 | 164.8 | ± | 19.4 | * | 118.1 | ± | 22.6 | 454.5 | ± | 47.7 | * |
| 8 | 140.5 | ± | 18.3 | 27.3 | ± | 36.6 | * | 77.1 | ± | 54.8 | 0.2 | ± | 0.2 | * |
| 9 | 46.8 | ± | 6.1 | 9.1 | ± | 12.2 | * | 25.7 | ± | 18.3 | 0.1 | ± | 0.1 | * |
| 10 | 275.8 | ± | 38.4 | 441.0 | ± | 142.1 |  | 256.3 | ± | 44.8 | 285.5 | ± | 111.3 |  |
| 11 | 270.4 | ± | 44.4 | 233.1 | ± | 57.9 |  | 182.8 | ± | 29.9 | 65.2 | ± | 38.0 | * |
| 12 | 2.5 | ± | 2.5 | 231.2 | ± | 114.8 |  | 81.2 | ± | 45.1 | 246.1 | ± | 109.7 | * |
| 13 | 41.1 | ± | 9.5 | 310.7 | ± | 127.4 |  | 113.1 | ± | 66.8 | 254.3 | ± | 108.9 | * |
| 14 | 106.2 | ± | 8.8 | 373.4 | ± | 141.6 |  | 130.0 | ± | 66.4 | 268.2 | ± | 108.8 | * |
| 15 | 108.7 | ± | 10.5 | 380.9 | ± | 147.4 |  | 130.0 | ± | 66.4 | 268.4 | ± | 108.8 | * |
| 16 | 5.4 | ± | 6.0 | 207.8 | ± | 88.7 |  | 73.5 | ± | 45.4 | 220.3 | ± | 105.1 | * |
| 17 | 234.7 | ± | 30.3 | 135.9 | ± | 24.0 | * | 143.2 | ± | 45.8 | 31.2 | ± | 16.3 | * |
| 18 | 22.2 | ± | 6.9 | 0.1 | ± | 0.1 | * | 8.2 | ± | 20.1 | 1.6 | ± | 3.9 |  |
| 19 | 125.4 | ± | 14.4 | 173.2 | ± | 59.0 |  | 64.7 | ± | 12.0 | 49.7 | ± | 21.7 |  |
| 20 | 38.6 | ± | 11.8 | 73.9 | ± | 25.8 |  | 31.9 | ± | 24.8 | 8.2 | ± | 9.2 |  |
| 21 | 212.5 | ± | 34.9 | 135.8 | ± | 24.0 | * | 135.0 | ± | 40.1 | 29.6 | ± | 17.6 | * |
| 22 | 498.5 | ± | 61.2 | 1184.2 | ± | 524.5 |  | 453.3 | ± | 159.0 | 596.4 | ± | 448.5 |  |
| 23 | 224.5 | ± | 11.8 | 475.0 | ± | 159.9 |  | 158.9 | ± | 65.6 | 292.2 | ± | 108.8 | * |
| 24 | 29.3 | ± | 3.8 | 17.0 | ± | 3.0 | * | 17.9 | ± | 5.7 | 3.9 | ± | 2.0 | * |
| 25 | 31.1 | ± | 2.2 | 16.1 | ± | 2.2 | * | 17.0 | ± | 2.1 | 10.8 | ± | 1.9 | * |
| 26 | 21.6 | ± | 1.8 | 10.4 | ± | 1.5 | * | 11.3 | ± | 0.6 | 9.6 | ± | 1.3 | * |
| 27 | 9.4 | ± | 1.0 | 5.6 | ± | 1.0 | * | 5.7 | ± | 1.9 | 1.2 | ± | 0.7 | * |
| 28 | 46.1 | ± | 1.8 | 41.4 | ± | 7.6 |  | 12.3 | ± | 0.5 | 11.0 | ± | 1.0 | * |
| 29 | 5.7 | ± | 4.0 | 0.3 | ± | 0.3 |  | 0.0 | ± | 0.1 | 0.3 | ± | 0.3 |  |
| 30 | 99.7 | ± | 6.5 | 85.8 | ± | 18.5 |  | 22.1 | ± | 3.4 | 15.2 | ± | 8.1 |  |
| 31 | 19.1 | ± | 6.3 | 42.2 | ± | 6.0 | * | 0.2 | ± | 0.1 | 0.6 | ± | 0.3 | * |
| 32 | 4.3 | ± | 0.3 | 3.9 | ± | 0.4 |  | 1.5 | ± | 0.1 | 0.0 | ± | 0.0 | * |
| 33 | 0.0 | ± | 0.0 | 0.0 | ± | 0.0 |  | 0.0 | ± | 0.0 | 0.0 | ± | 0.0 |  |
| 34 | 36.5 | ± | 21.5 | 2.7 | ± | 2.4 |  | 6.8 | ± | 16.7 | 9.2 | ± | 21.9 |  |
| 35 | 0.0 | ± | 0.0 | 5.6 | ± | 5.4 |  | 0.0 | ± | 0.0 | 0.0 | ± | 0.0 |  |
| 36 | 20.2 | ± | 9.7 | 9.8 | ± | 17.0 |  | 4.7 | ± | 1.3 | 0.0 | ± | 0.0 | * |
| 37 | 21.6 | ± | 1.8 | 16.5 | ± | 6.8 |  | 2.8 | ± | 0.4 | 2.6 | ± | 0.2 |  |
| 38 | 10.3 | ± | 5.0 | 13.4 | ± | 23.1 |  | 2.4 | ± | 0.7 | 0.3 | ± | 0.1 | * |
| 39 | 44.5 | ± | 1.7 | 40.5 | ± | 6.2 |  | 11.0 | ± | 0.5 | 9.4 | ± | 0.8 | * |
| 40 | 47.7 | ± | 1.9 | 41.3 | ± | 7.2 |  | 11.9 | ± | 0.6 | 9.9 | ± | 0.9 | * |
| 41 | 0.0 | ± | 0.0 | 0.2 | ± | 0.4 |  | 0.0 | ± | 0.0 | 0.0 | ± | 0.0 |  |
| 42 | 5.7 | ± | 4.0 | 0.3 | ± | 0.3 |  | 0.0 | ± | 0.1 | 0.3 | ± | 0.3 |  |
| 43 | 0.1 | ± | 0.1 | 2.7 | ± | 2.4 |  | 0.0 | ± | 0.0 | 0.0 | ± | 0.0 |  |
| 44 | 1.1 | ± | 1.0 | 2.4 | ± | 4.1 |  | 0.0 | ± | 0.0 | 0.0 | ± | 0.0 |  |
| 45 | 4.8 | ± | 2.0 | 10.8 | ± | 0.8 | * | 0.0 | ± | 0.0 | 1.3 | ± | 0.1 | * |
| 46 | 0.0 | ± | 0.0 | 4.6 | ± | 5.3 |  | 0.0 | ± | 0.0 | 0.0 | ± | 0.0 |  |
| 47 | 0.0 | ± | 0.0 | 1.1 | ± | 1.8 |  | 1.7 | ± | 0.2 | 2.6 | ± | 0.3 | * |
| 48 | 5.7 | ± | 4.0 | 0.1 | ± | 0.1 |  | 0.0 | ± | 0.0 | 0.3 | ± | 0.3 |  |
| 49 | 2.5 | ± | 2.2 | 7.6 | ± | 7.1 |  | 0.0 | ± | 0.0 | 0.2 | ± | 0.1 | * |
| 50 | 20.4 | ± | 1.0 | 19.5 | ± | 6.6 |  | 5.9 | ± | 0.2 | 4.4 | ± | 0.4 | * |
| 51 | 1.1 | ± | 0.9 | 0.1 | ± | 0.2 |  | 0.8 | ± | 0.2 | 0.4 | ± | 0.1 | * |
| 52 | 2.4 | ± | 1.1 | 4.8 | ± | 5.6 |  | 2.1 | ± | 2.6 | 6.1 | ± | 6.5 |  |
| 53 | 24.8 | ± | 2.4 | 42.3 | ± | 5.9 | * | 0.3 | ± | 0.1 | 0.9 | ± | 0.1 | * |
| 54 | 4.3 | ± | 0.3 | 3.9 | ± | 0.4 |  | 1.5 | ± | 0.1 | 0.0 | ± | 0.0 | * |
| 55 | 36.5 | ± | 21.4 | 0.0 | ± | 0.1 |  | 6.8 | ± | 16.7 | 9.2 | ± | 21.9 |  |
| 56 | 14.6 | ± | 3.0 | 12.9 | ± | 0.6 |  | 2.3 | ± | 0.6 | 1.0 | ± | 0.1 | * |
| 57 | 21.6 | ± | 1.8 | 17.6 | ± | 5.6 |  | 4.5 | ± | 0.3 | 5.3 | ± | 0.4 | * |
| 58 | 21.7 | ± | 1.6 | 23.8 | ± | 1.5 |  | 2.8 | ± | 0.4 | 2.6 | ± | 0.2 |  |
| 59 | 1.4 | ± | 1.2 | 5.2 | ± | 4.6 |  | 0.0 | ± | 0.0 | 0.2 | ± | 0.1 | * |
| 60 | 384.9 | ± | 51.1 | 847.3 | ± | 355.0 |  | 306.2 | ± | 111.9 | 445.7 | ± | 278.7 |  |
| 61 | 558.6 | ± | 85.9 | 1146.3 | ± | 385.3 |  | 570.7 | ± | 137.4 | 804.1 | ± | 328.3 |  |
| 62 | 124.2 | ± | 30.5 | 82.4 | ± | 29.2 |  | 31.6 | ± | 16.7 | 33.2 | ± | 21.0 |  |
| 63 | 546.1 | ± | 82.8 | 674.1 | ± | 198.0 |  | 439.1 | ± | 61.1 | 350.8 | ± | 128.9 |  |
| 64 | 273.3 | ± | 40.9 | 209.8 | ± | 48.0 |  | 175.1 | ± | 31.8 | 39.4 | ± | 24.3 | * |
| 65 | 38.6 | ± | 11.8 | 79.5 | ± | 22.7 |  | 31.9 | ± | 24.8 | 8.2 | ± | 9.2 |  |
| 66 | 103.3 | ± | 16.4 | 173.1 | ± | 59.0 |  | 56.5 | ± | 23.0 | 48.1 | ± | 22.7 |  |

All units are in mmol/g-DNA/2 days. Reaction numbers refer to Table S1. Data shown are means ± SD (*n* = 3 for pRev and UCP1; *n* = 6 for untreated control and FCCP). Statistical tests compared UCP1 against pRev and FCCP against untreated control.*: Significantly different from pRev or untreated control (p < 0.05).
