# Supplementary material for: Metabolic Flux Analysis of Mitochondrial Uncoupling in 3T3-L1 Adipocytes
Source: PLoS One. 2009 Sep 10;4(9):e7000. doi: 10.1371/journal.pone.0007000 (PMC2734990; doi:10.1371/journal.pone.0007000)
Supplement: Table S3 — Effect of forced UCP1 expression on cellular ATP (0.03 MB DOC) [file pone.0007000.s006.doc]

**Table S3**. Effect of forced UCP1 expression on cellular ATP.

| **Time, hr** | **Starved** | | | | | | | **Fed** | | | | | | |
| --- | --- | --- | --- | --- | --- | --- | --- | --- | --- | --- | --- | --- | --- | --- |
|  | **pRev** | | | **UCP1** | | | | **pRev** | | | **UCP1** | | | |
| 0 | 6.25 | ± | 0.38 | 6.27 | ± | 0.55 |  | 6.25 | ± | 0.38 | 6.27 | ± | 0.55 |  |
| 48 | 3.49 | ± | 0.28 | 3.08 | ± | 0.29 | * | No Data | | | No Data | | |  |
| 72 | 4.44 | ± | 0.34 | 3.49 | ± | 0.24 | * | No Data | | | No Data | | |  |
| 96 | 3.51 | ± | 0.36 | 3.16 | ± | 0.37 |  | 5.84 | ± | 0.61 | 6.27 | ± | 0.44 |  |

As in Figure 3, time zero corresponds to day 10 post-induction and glucose starvation lasted for 48 hrs. Cells were again fed glucose-rich (4.5 g/L) maintenance medium at 48 hrs. Data shown are means ± SD (*n* = 6). Statistical tests compared UCP1 against pRev at the same time point.*: Significantly different from pRev (p < 0.05).
